# Near-infrared spectroscopy StO_2_ monitoring to assess the therapeutic effect of drotrecogin alfa (activated) on microcirculation in patients with severe sepsis or septic shock

**DOI:** 10.1186/2110-5820-3-30

**Published:** 2013-09-04

**Authors:** Jordi Masip, Jaume Mesquida, Cecilia Luengo, Gisela Gili, Gemma Gomà, Ricard Ferrer, Jean Louis Teboul, Didier Payen, Antonio Artigas

**Affiliations:** 1 Critical Care Department, Hospital de Sabadell, CIBER Enfermedades Respiratorias, Consorci Sanitari Universitari Parc Tauli, Universitat Autònoma de Barcelona, Parc Taulí s/n, Sabadell (Barcelona) CP 08208, Spain; 2 Department of Medicine, Intensive Care Unit, Critical Care Patient Unit, Hospital Clínico Universidad de Chile, Avenida Santos Dumontt 999, Independencia, Santiago, Chile; 3 Department of Anesthesiology & Critical Care Medicine, SAMU and Laboratory of Anesthesiology, Hospital Lariboisière, Paris, 2, rue Ambroise – Paré, 75010 Paris, 10ème, France; 4 Intensive Care Department, Mutua Terrassa University Hospital, University of Barcelona. CIBER Enfermedades Respiratorias, Plaça Doctor Robert, Terrassa (Barcelona) CP: 08221, Spain; 5 Service de Réanimation Médicale, Centre Hospitalo-Universitaire de Bicêtre, 78 rue du Général-Leclerc, Le Kremlin-Bicêtre 94 270, France

**Keywords:** Severe sepsis, Septic shock, Tissue oxygen saturation, Near-infrared spectroscopy, Drotrecogin alfa activated, Outcome

## Abstract

**Background:**

Sepsis is a leading cause of death despite appropriate management. There is increasing evidence that microcirculatory alterations might persist independently from macrohemodynamic improvement and are related to clinical evolution. Future efforts need to be directed towards microperfusion monitoring and treatment. This study explored the utility of thenar muscle oxygen saturation (StO_2_) and its changes during a transient vascular occlusion test (VOT) to measure the microcirculatory response to drotrecogin alfa (activated) (DrotAA) in septic patients.

**Methods:**

A prospective, observational study was performed in three general intensive care units at three university hospitals. We studied 58 patients with recent onset of severe sepsis or septic shock and at least two organ dysfunctions. Thirty-two patients were treated with DrotAA and 26 were not treated because of formal contraindication. StO_2_ was monitored using near-infrared spectroscopy (NIRS), and VOT was performed to obtain deoxygenation (DeOx) and reoxygenation (ReOx) slopes. Measurements were obtained before DrotAA was started and were repeated daily for a 96-hour period.

**Results:**

Patients’ characteristics, outcome, severity, and baseline values of StO_2_, DeOx, and ReOx did not differ between groups. Treated patients significantly improved DeOx and ReOx values over time, whereas control patients did not. In treated patients, ReOx improvements were correlated to norepinephrine dose reductions. Early clinical response (SOFA improvement after 48 hours of treatment) was not associated to changes in VOT-derived slopes. In the treated group, the relative improvement of DeOx within 48 hours was able to predict mortality (AUC 0.91, *p* < 0.01).

**Conclusions:**

In patients with severe sepsis or septic shock, DrotAA infusion was associated with improvement in regional tissue oxygenation. The degree of DeOx amelioration after 2 days in treated patients predicted mortality with high sensitivity and specificity. Thus, StO_2_ derived variables might be useful to evaluate the microcirculatory response to treatment of septic shock.

## Background

Sepsis is a leading cause of death in critically ill patients despite the use of modern antibiotics and resuscitation strategies [1]. In recent years, the introduction of early goal-directed therapy (EGDT) has improved outcome of severe sepsis and septic shock [2,3]. EGDT interventions seek the normalization of global markers of hypoperfusion, such as lactate and venous oxygen saturations (central or mixed). However, sepsis is a complex syndrome that affects the microcirculation and tissue microperfusion. There is increasing evidence that persistent microcirculatory alterations are related to clinical evolution independently from macrohemodynamic improvement [4-8]. Increased microvascular heterogeneity during sepsis mediated by the presence in varying degrees of stopped-flow capillaries, cell aggregation and thrombosis phenomena, vasoconstriction, and alteration of red cell flexibility among others [9] might be only partially reversed by macrohemodynamics resuscitation strategies. This strengthens the opinion that future research efforts need to be directed towards monitoring technologies and therapies targeting tissue microperfusion.

Near-infrared spectroscopy (NIRS) technology measures tissue hemoglobin oxygen saturation (StO_2_) in a tissue sample volume containing the three vascular compartments (arterial, capillary, and venous). Tissue perfusion impairment increases O_2_ extraction, and thus, it decreases venous O_2_ saturation, resulting in lower StO_2_ values. In clinical scenarios, StO_2_ has been associated with patients’ alterations in peripheral circulation [10], as well as in healthy volunteers [11], it has shown its ability to predict noninvasively the presence of global tissue hypoxia [12-14] and has demonstrated prognostic utility [15,16]. For instance, persistent low StO_2_ values have been related to worse organ failure evolution either in septic shock [16] or hemorrhagic shock [15]. Thus, improvement in tissue perfusion might be reflected by StO_2_ monitoring. A dynamic functional test consisting in a transient vascular occlusion (VOT) seems to improve the assessment of the microperfusion. According to the StO_2_ response to ischemia and reperfusion, StO_2_ deoxygenation (DeOx) and reoxygenation (ReOx) rates are obtained. Local tissue O_2_ consumption can be evaluated by analyzing the DeOx slope during the period of ischemia, and the ReOx following cuff-release reflects the ability of the tissue to recruit oxygenated blood volume, depending on both endothelial integrity and perfusion pressure. Importantly, VOT-derived variables have been independently associated to peripheral circulation derangements [10], and both, DeOx and ReOx have shown their prognostic value consistently in septic conditions [17-22].

Effect of DrotAA infusion of the microcirculatory alterations has been evaluated either using direct measures of microperfusion, such as video-microscopy, or using tissue oxygenation measurements, such as StO_2_-derived variables [23,24]. Because StO_2_-derived parameters have been proven useful to detect the DrotAA effects on microperfusion [24], we hypothesized that differences in StO_2_-derived parameters evolution might reflect differences in microperfusion among patients whose microcirculation responds to DrotAA and those that do not respond. In that case, StO_2_ monitoring might be useful to guide therapy.

## Methods

The study was designed as a prospective, observational research in three intensive care units (ICU). The three ICUs were from Hôpital de Bicêtre and Hôpital Lariboisière in France and Hospital de Sabadell in Spain. The Ethical Review Board of each participating center approved the study, and oral or written informed consent was obtained from the patient or next of kin according to the local review board’s requirement.

### Patients

Patients were recruited according to the following inclusion criteria: age >18 years old, with proven or suspected infection; infection was suspected when white blood cells were present in a normally sterile body fluid, or a viscera was perforated, or purulent sputum production was present associated with chest x-ray consistent with pneumonia, or a clinical syndrome of inflammation was associated with a high probability of infection, such as purpura fulminans or ascending cholangitis; presence of severe sepsis [25], with at least two sepsis-associated organ failures, newly developed (within the 24 hours before enrollment), and not explained by an underlying disease or by the effects of concomitant therapy.

Among the patients who met inclusion criteria two groups were defined: the treated group who received DrotAA and the control group, which did not receive this therapy because of formal contraindication. Formal contraindication to receive DrotAA was considered when one or more of the following criteria were present: platelet count <30,000/mm^3^, active bleeding or increased risk of bleeding, need for therapeutic heparin, warfarin, or other anticoagulants, antiplatelets or glycoprotein IIb/IIIa receptor antagonists, thrombolytic therapy, antithrombin, or recombinant factor VIIa therapy.

#### *Exclusion criteria*

Patients were excluded: when they declined to give their written, informed consent; those who have a body weight <30 kg or >135 kg; a documented multiple organ dysfunction for more than 24 hours before inclusion; a delay >36 hours before the start of DrotAA for the first organ dysfunction; died or being moribund (within 24 hours); HIV/AIDS and known end-stage processes; attended by staff not committed to aggressive management; pregnant or lactating women; trauma in both upper limbs, or haematoma or skin lesions in the thenar eminence that could hinder placement of the NIRS sensor probe.

During data acquisition, no new concomitant medications were prescribed. Standard treatment was applied following local clinical protocols according to the Surviving Sepsis Campaign recommendations [3]. Patients in the treated group received a continuous infusion of DrotAA (24 μg/kg/hour) for 96 hours.

### Measurements

Patient demographics, diagnosis at ICU admission, focus of sepsis, and Acute Physiology and Chronic Health Evaluation II (APACHE II) and Simplified Acute Physiology Score II (SAPS II) scores were recorded at inclusion.

### Thenar oxygen saturation (StO_2_)

Thenar StO_2_ was measured using an InSpectra™ StO_2_ Monitor model 650 or model 325 (Hutchinson Technology Inc; Hutchinson, MN) with a 15-mm probe, according to device availability at each site. The probe was placed on the thenar eminence, on intact skin, and never adjacent to an arterial line.

The VOT was performed using a pneumatic cuff tourniquet placed around the forearm, proximal to the StO_2_ probe, as previously described [26]. Patients remained in a semirecumbent position, with their arms straight on the bed. The cuff-pressure was raised quickly (3–5 seconds) up to 40 mmHg above systolic blood pressure. According to local VOT protocol, vascular occlusion was stopped either once the StO_2_ value dropped to 40% or after 3 minutes of ischemia. Once the cuff was deflated, StO_2_ was recorded until its value remained stable again.

Baseline steady-state StO_2_, DeOx, and ReOx were automatically computed using the InSpectra Analysis Software provided by Hutchinson Technology Inc.

### Follow-up

The following data were obtained and recorded at inclusion (T0) and every 24 hours up to 96 hours, obtaining five sets of measurements (T0, T24, T48, T72, and T96):

• NIRS-derived variables: StO_2_, DeOx, and ReOx.

• Hemodynamic, respiratory, and blood sample parameters: arterial blood pressure, heart rate, vasoactive drug doses, pulse-oximeter oxygen saturation (SpO_2_), ventilator settings if intubated, arterial and central venous blood gases and acid–base status, lactate, hemoglobin, and axillary temperature.

• Sequential Organ Failure Assessment (SOFA) score.

In order to analyze the early clinical response of the DrotAA group, patients whose SOFA score decreased ≥25% at T48 were classified as early SOFA-responders. Mortality at day 28 and ICU length of stay also were recorded.

### Statistical analysis

Data were analyzed using PASW version 18 (IBM–SPSS; Chicago, IL). Descriptive statistics were computed for all the studied variables. Continuous variables were expressed as mean ± SD, and categorical variables were reported as proportions (%). We used two-tailed Pearson’s chi-square tests to compare categorical variables between groups and two-tailed Mann–Whitney *U* tests to compare continuous variables. Wilcoxon test for repeated measurements was used to evaluate the time course of the StO_2_ variables. Spearman’s rho test was used to evaluate the correlation among NIRS-derived variables with SOFA, mean arterial pressure (MAP), and norepinephrine (NE) dose evolution. Receiver operating characteristic test (ROC curve) was performed to analyze predictive potential of NIRS-derived variables. All statistics were two-tailed, and *p* < 0.05 was considered to be significant.

## Results

A total of 58 patients with severe sepsis or septic shock were included: 32 patients treated with DrotAA (treated group), and 26 patients who did not receive DrotAA (control group). The main clinical characteristics and outcomes for each group of patients are shown in Table 1. Treated patients were younger than control patients (63 ± 15 vs. 73 ± 12 years, *p* < 0.02) and received higher baseline NE doses (0.8 ± 1.0 μg/kg/min vs. 0.4 ± 0.3 μg/kg/min, *p* = 0.03). No differences in other baseline hemodynamic, respiratory, metabolic, and tissue hemoglobin saturation parameters were found between the two groups.

**Table 1:** Clinical characteristics of the studied patients

		**Treated (n = 32)**	**Controls (n = 26)**	***p***
Gender (male %)		63%	50%	0.3
Age (yr)		63 ± 15	73 ± 12	**0.02**
Origin of sepsis				
Abdominal		56%	42%	
Bone		0%	4%	
Respiratory		34%	27%	
Skin/soft tissue		10%	23%	
Urinary tract		0%	4%	
SAPS II		56 ± 13	59 ± 11	0.4
APACHE II		25 ± 6	24 ± 7	0.6
SOFA score at inclusion		11 ± 3	11 ± 2	0.4
Value at inclusion	Mean arterial pressure (mmHg)	77 ± 9	75 ± 11	0.6
	Norepinephrine dose (mcg/kg/min)	0.8 ± 1.0	0.4 ± 0.33	**0.04**
	Heart rate (bpm)	94 ± 20	96 ± 22	0.7
	Lactate (mmol/L)	3.5 ± 2.0	4.0 ± 3.5	0.6
	pH	7.30 ± 0.11	7.31 ± 0.12	0.8
	Base deficit (mmol/L)	−9.0 ± 4.3	−7.5 ± 7.2	0.4
	ScvO_2_ (%)	70 ± 10	71 ± 13	0.8
	SpO_2_ (%)	96 ± 4	98 ± 3	0.2
	PaO_2_/FiO_2_	181 ± 102	255 ± 164	0.3
	Hb (g/dL)	11.2 ± 2.0	10.9 ± 2.2	0.6
	StO_2_ (%)	80 ± 8	78 ± 12	0.5
	Deoxygenation slope (%/min)	−16.2 ± 18	−17.4 ± 14.4	0.4
	Reoxygenation slope (%/s)	3.2 ± 2.6	2.3 ± 1.8	0.1
Mortality (n, %)		6 (19)	10 (38)	0.09
Length of ICU stay (days)		26 ± 21	19 ± 17	0.2

Clinical characteristics at inclusion according to treatment group. Continuous variables are expressed as mean ± SD. Categorical variables are expressed as proportions (%). Two-tailed significance is shown under “*p*” column.

### Hemodynamics and organ failure evolution

**Figure 1:**
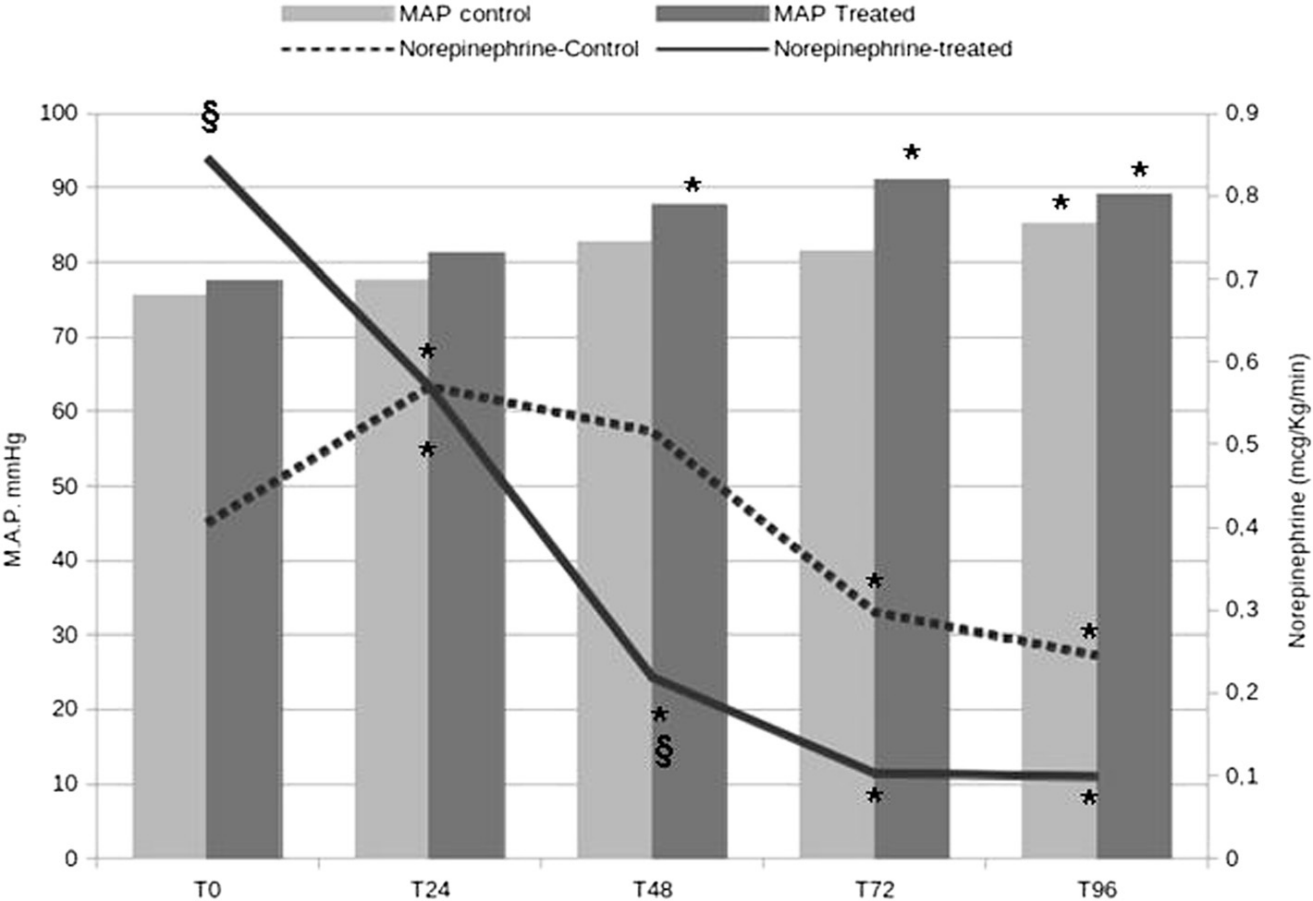
**Evolution over time in treated and control patients.** *p < 0.05 compared with T_0_; ^**§**^*p* < 0.05 compared with control patients.

Treated patients significantly increased their MAP after 48 hours of treatment, whereas in control patients MAP only improved at T96 (Figure 1). NE infusion evolution also was different in both groups. The treated group showed a significant decrease in NE infusion rate at each time point of the study, whereas the control group showed a significant increase in NE requirements at T24, with progressive reduction of NE doses starting at T48 (Figure 1).

The evolution of organ failure assessed by SOFA score was similar between groups: 34% of patients were considered to be early SOFA-responders in the treated group, and 19% in the control group. No differences in SOFA score at each time point of the study were found between treated and control patients. However, treated patients showed significantly earlier SOFA decrease at T72 and T96 (*p* < 0.01 in both cases), whereas in control patients SOFA decreased significantly only at T96 (*p* < 0.01). Overall, mortality rate was 28% (19% in the treated group and 38% in the control group, *p* = 0.09).

### StO_2_ evolution: treated vs. control

**Figure 2:**
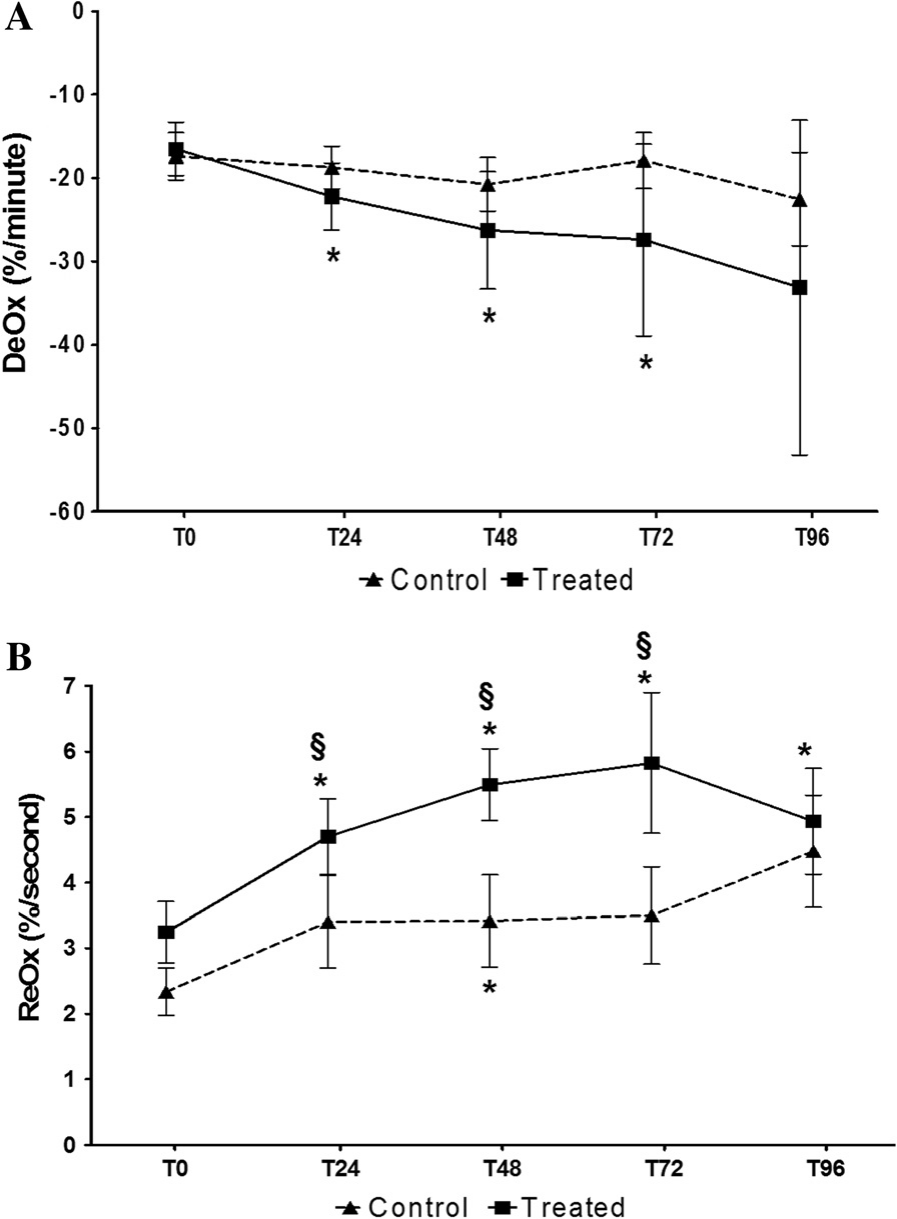
**StO2-derived variables evolution: treated vs control group. A.** DeOx evolution in treated and control patients. Mean and SE are represented. *p < 0.05 compared with T_0_; ^**§**^*p* < 0.05 compared with control patients. **B.** ReOx evolution in treated and control patients. Mean and SE are represented. *p < 0.05 compared with T_0_; ^**§**^*p* < 0.05 compared with control patients.

Absolute steady-state StO_2_ did not change over time, and no differences in its evolution were observed when comparing treated and control groups. In the treated group, DeOx and ReOx values improved significantly and consistently over time, at each time point of the study. This evolution was not observed in control patients, in whom DeOx remained unchanged (Figure 2A), and ReOx only increased at T48 (Figure 2B). Relative improvement in DeOx over time was not correlated to relative changes in MAP or NE use over time in both groups. However, in treated patients, changes in ReOx over time were inversely correlated to relative changes in NE dosage over time (r = −0.5, *p* < 0.01 at T24; r = −0.5, *p* < 0.02 at T48) but not to MAP changes. This association was not observed in the control group.

### DrotAA patients: organ failure evolution and mortality

**Figure 3:**
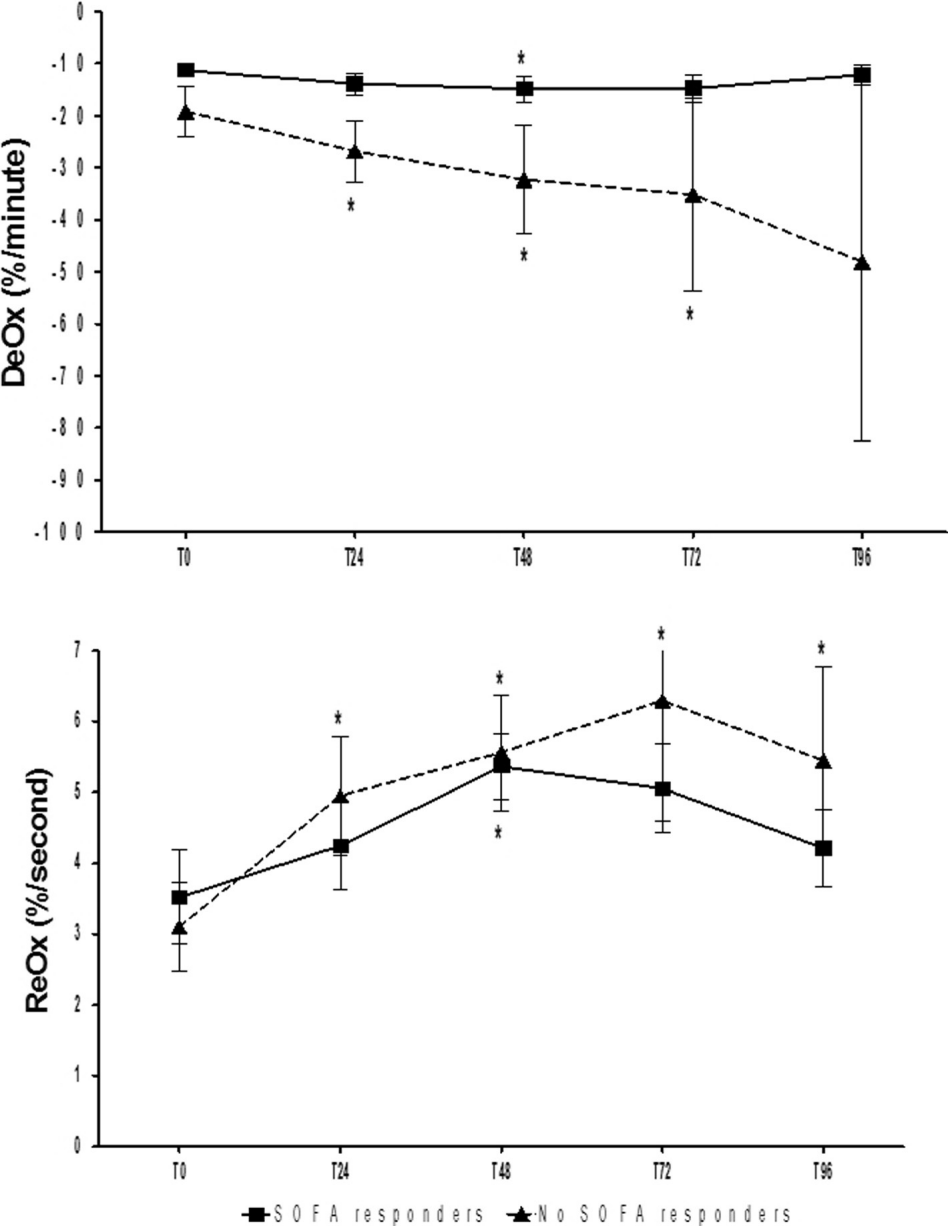
**None of the VOT-derived variables was associated with early SOFA improvement.** Mean and SE are represented. *p < 0.05 compared with T_0_.

**Figure 4:**
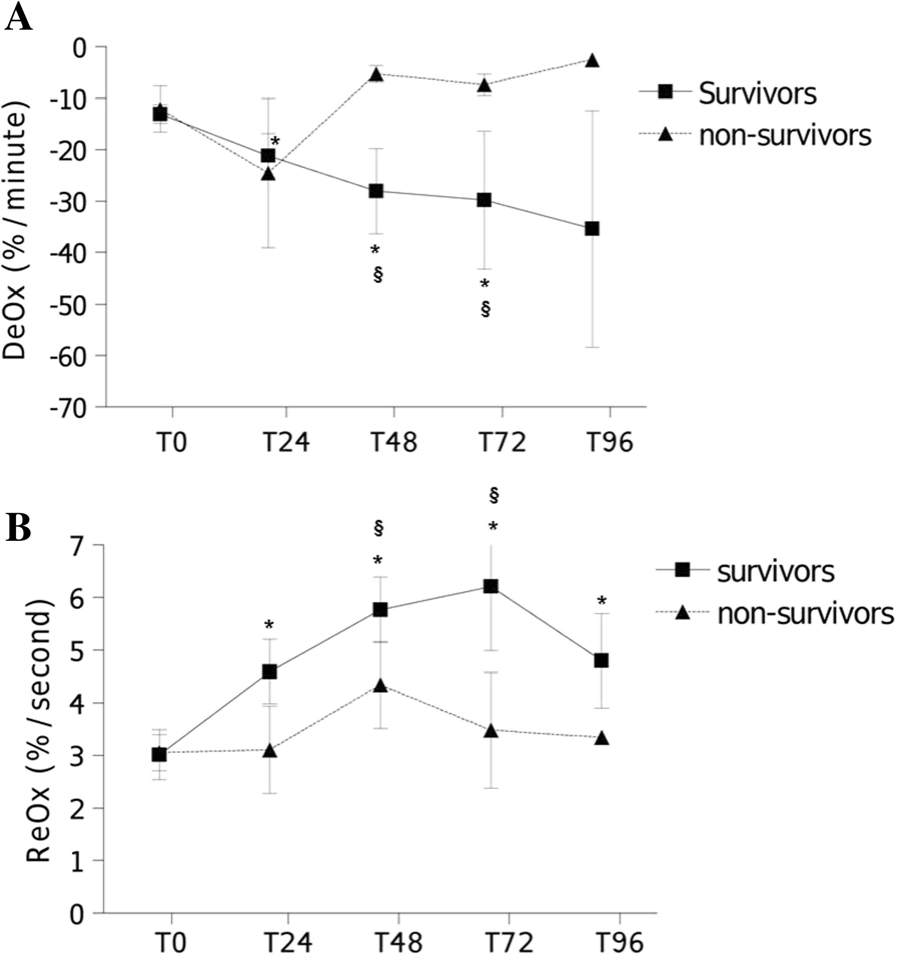
**StO2-derived variables evolution and outcome. A.** DeOx evolution according to mortality. Mean and SE are represented. *p < 0.05 compared with T_0_; ^**§**^*p* < 0.05 compared with nonsurvivors. **B.** ReOx evolution according to mortality. Mean and SE are represented. *p < 0.05 compared with T_0_; ^**§**^*p* < 0.05 compared with nonsurvivors.

When analyzing treated patients, similar patterns in StO_2_-variables evolution were observed according to organ failure evolution. Clinical responders and nonresponders at T48 (SOFA48 5 ± 1 responders vs. 12 ± 3 nonresponders, *p* < 0.01) showed no significant differences in StO_2_, DeOx, and ReOx evolution compared with nonresponders (Figure 3). However, DeOx and ReOx evolution significantly differed when comparing patients according to 28-day mortality (Figure 4). Both DeOx and ReOx significantly improved over time in patients who survived, whereas no time-related change was observed in patients who did not. The relative improvement in DeOx, measured as the ratio between DeOx48 and DeOx0, was the best parameter for 28-day mortality prediction, with an AUC of 0.91 (*p* < 0.01).

## Discussion

In the present study, we sought to confirm the capacity of StO_2_-derived variables during DrotAA infusion to assess the response of the microcirculation to this treatment. We analyzed whether the evolution of these variables would predict clinical evolution and outcome. Because absolute steady-state StO_2_ has consistently revealed poor sensitivity to detect regional hypoperfusion in septic patients [12,22,27], we did not expect to find any differences in this parameter between treated and control patients, neither between early SOFA-responders and SOFA-nonresponders in the treated group. Indeed, our data support the lack of sensitivity of this parameter when measured on the thenar eminence.

In our population, we confirmed a different evolution of VOT-derived variables associated to DrotAA therapy. Previous observations [24] are corroborated in our multicenter cohort, with the detection of progressive local oxygen consumption improvements, as suggested by the DeOx response, associated to DrotAA therapy.

ReOx improvements also have been found. Considering the simultaneous evolution patterns of MAP, NE requirements, and ReOx improvement, it might suggest that the DrotAA group displayed an earlier recovery of the vascular endothelial function compared with control patients. Regrettably, despite both groups were managed using the same clinical protocols, as far as other interventions that might affect the microcirculation were not computed (such as red blood cell transfusion), we cannot attribute this improvement to DrotAA therapy. Nevertheless, ReOx improvements may account for the significant reductions observed in NE requirements to maintain similar or even higher MAP levels. Therefore, our results suggest that ReOx would be able to reflect endothelial integrity improvement, as its increases matched NE reductions.

Many studies have underscored the prognostic implications of either DeOx or ReOx [16-22]. In our data, DeOx evolution was not associated to early clinical improvement, as measured by SOFA changes at T48. However, in our treated population, the degree of improvement in DeOx was directly associated with survival, whereas in control patients DeOx response did not change over time nor was associated to outcome. The interpretation of these differences of association is complex: our first elucidation would be that although we were unable to detect early recovery of organ failure, being able to recruit the microcirculation might have impact on survival. First, although microcirculatory impairment might be the initial fundamental condition for organ dysfunction development, organ recovery might not parallel microcirculatory changes. Other conditions, such as mitochondrial dysfunction, might play an important role in the recuperation of cellular function. Second, we also need to take into account the potential limitations of the score used for organ dysfunction measurement. It seems obvious that SOFA score might not be sensitive enough to detect slight variations in organ function. Finally, the fact that these associations were not present in the control group also could also mean that DeOx variation is just an associated phenomenon to DrotAA therapy that does not change clinical evolution.

In our population, despite ReOx significantly improved over time in the treatment group, the magnitude of ReOx augmentation was not associated with clinical evolution or mortality. Since both, endothelial integrity and tissue perfusion pressure might account for the final observed ReOx value [22,28], the interpretation of this variable appears complex. Although ReOx has demonstrated its predictive value in many studies, it seems to be subjected to several factors that could act as confounding factors, which might limit its use for therapy guidance purposes.

Taking into account these contradictory results, the fact that DrotAA patients clearly improved regional oxygenation, as previously shown by others [23,24], but DrootAA has failed repeatedly to prove effectiveness on prognosis [29,30], lead us to the next questions: Are microcirculation-targeted therapies futile? Despite our results support the DrotAA effect on regional tissue perfusion, the degree of regional improvement derived from this therapy might not be associated to better prognosis. Furthermore, similar StO_2_ evolution patterns have been noticed for all treated patients, regardless of their clinical evolution. Furthermore, initial StO_2_ parameters were not able to predict StO_2_ response to therapy, and thus, it could not have been used to decide whether to treat, further limiting the use of StO_2_ in the decision tree. Finally, it should be considered that the observed results might be an epiphenomena with no clinical implications. Further studies are needed to clarify those points, as the present study has several limitations that prevent this question to be elucidated.

### Study limitations

A major limitation, that restrains the interest of the results, is that it provides data about a therapy that is no longer available due to its lack of proven effectiveness. Although StO_2_ derived variables have demonstrated their ability to detect the effects of DrotAA on microcirculation, it has not been proven that these changes have repercussion on outcome. Nevertheless, these results are interesting as they prove the utility of a noninvasive monitoring system to follow microcirculation response to a given therapy and opens new opportunities to study the effects new therapies on microcirculation of other therapies, the impact of microcirculation amelioration on outcome, and to develop treatment tailoring strategies, not possible until now due to a lack of available method to evaluate individual response easily.

The second major limitation is that although prospective and multicentric, this study has some important issues to take into account. First, the treated and control group differed for some clinical characteristics; age and intensity of cardiovascular support. Second, the observational nature of the study implied that the control group consisted in patients with contraindications for DrotAA therapy, which might differ from a real matched control group. We cannot exclude that the different age or previous pathologies that contraindicated DrotAA therapy could explain some of the observed differences. Nevertheless, the severity scores were similar and the dose of norepinephrine required for maintaining the targeted MAP at the time of inclusion was even higher in the treated group, suggesting that the patients of the treated group were not less sick than the patients of the control group. Third, the impact of other supportive therapies, such as fluid resuscitation or blood transfusion on StO_2_ and clinical evolution, was not analyzed. Although the participating centers followed the Surviving Sepsis Campaign guidelines, differences in administered therapies might have occurred. Fluid resuscitation is one major point of the Surviving Sepsis Campaign guidelines and has direct impact on microcirculation. The guidelines remark the importance of early fluid administration, but the timespan of the present study goes farther than the early phase. Because many global hemodynamic parameters are used for fluid-administration guidance, and the microcirculation has shown repeatedly its uncoupling from macrocirculation within the time-course of sepsis [31,32], it is difficult to interpret the relationship between appropriate fluid resuscitation and microcirculation. The definition of “appropriate” fluid administration, both in early and late phase of sepsis management, is not an easy task, and we are still far from integrating macro- and microcirculation for evaluation of fluid optimization purposes.

Hence, tissue oxygenation improvement cannot be solely attributed to DrotAA therapy. However, our results emphasize that StO_2_-derived parameters might be clinically useful to evaluate microcirculation response to drugs or interventions in sepsis.

Finally, two different VOT protocols have been used, as StO_2_ monitoring was performed using the local protocol of each center. Fifty percent of treated patients and all control patients were evaluated using a time-guided protocol. Although the use of time-guided (3 minutes of occlusion) or StO_2_-guided (reaching an ischemic StO_2_-threshold of 40%) VOT protocol has no influence on StO_2_, or DeOx values, it may affect the ReOx response, as stated by some authors [33-35]. Therefore, there might be slight differences in ReOx among patients derived from the ischemic challenge methodology used. However, the fact that only 15% of the studied patients using time-guided VOT did not reach the StO_2_ ischemic threshold within 3 minutes, and that the evolution of StO_2_ parameters were analyzed using the same VOT protocol in each patient, minimized the impact of this methodological limitation.

## Conclusions

In a population of patients with severe sepsis or septic shock, DrotAA infusion was associated with improved regional tissue oxygenation over time, as quantified by DeOx and ReOx. The relative improvement in DeOx after 2 days of therapy predicted mortality with high sensitivity and specificity.

Although our results cannot establish a causality relationship between DrotAA infusion and tissue oxygenation improvement, they reinforce the fact that StO_2_-derived parameters might be useful to monitor the impact of therapy on the microcirculation, as well as to predict outcome. Assessment of microperfusion state and evolution in response to therapies targeting microcirculation should be evaluated further as a way to develop better and more individualized therapies.

## Abbreviations

APACHE II: Acute physiology and chronic health evaluation II; AUC: Area under curve; DeOx: Deoxygenation; DrotAA: Drotrecogin alfa (activated); EGDT: Early goal-directed therapy; MAP: Mean arterial pressure; NE: Norepinephrine; NIRS: Near-infrared spectroscopy; ReOx: Reoxygenation; ROC curve: Receiver operating characteristic test; SAPS II: Simplified acute physiology score II; ScvO2: Central venous oxygen saturation; SOFA score: Sequential organ failure assessment score; StO2: Tissue hemoglobin oxygen saturation; T0: inclusion time; T24: T48, T72, T96, Follow up measurement times at 24, 48, 72, and 96 hours after inclusion; VOT: Vascular occlusion test.

## Competing interests

The authors declare that they have no competing interests.

## Authors’ contributions

JM participated in the design of the study and collection and analysis of data and drafted the manuscript. JM participated in the analysis of data and drafted the manuscript. CL participated in the collection and processing of data and in the manuscript drafting. GG and GG participated in collection and processing of data. RF participated in the design of the study and revision of the manuscript. JLT participated in the design of the study, collection of data and revision of the manuscript. DP participated in design of the study and revision of the manuscript. AA participated in the design of the study, and revised the manuscript. All authors read and approved the final manuscript.
